# Distinct signatures of codon and codon pair usage in 32 primary tumor types in the novel database CancerCoCoPUTs for cancer-specific codon usage

**DOI:** 10.1186/s13073-021-00935-6

**Published:** 2021-07-28

**Authors:** Douglas Meyer, Jacob Kames, Haim Bar, Anton A. Komar, Aikaterini Alexaki, Juan Ibla, Ryan C. Hunt, Luis V. Santana-Quintero, Anton Golikov, Michael DiCuccio, Chava Kimchi-Sarfaty

**Affiliations:** 1 Hemostasis Branch, Division of Plasma Protein Therapeutics, Office of Tissues and Advanced Therapies, Center for Biologics Evaluation & Research, US Food and Drug Administration, Silver Spring, MD USA; 2 Department of Statistics, University of Connecticut, Storrs, CT USA; 3 Center for Gene Regulation in Health and Disease, Department of Biological, Geological and Environmental Sciences, Cleveland State University, Cleveland, OH USA; 4 Department of Anesthesiology, Critical Care and Pain Medicine, Boston Children’s Hospital and Harvard Medical School, Boston, MA USA; 5 High-performance Integrated Virtual Environment, Center for Biologics Evaluation and Research, Food and Drug Administration, Silver Spring, MD 20993 USA; 6 National Center of Biotechnology Information, National Institutes of Health, Bethesda, MD USA

**Keywords:** CancerCoCoPUTs, The Cancer Genome Atlas (TCGA), Cancer transcriptome, Codon usage, Codon pair, Relative synonymous codon usage (RSCU), Synonymous codons, Invasive ductal carcinoma, Invasive lobular carcinoma, Survival analysis

## Abstract

**Background:**

Gene expression is highly variable across tissues of multi-cellular organisms, influencing the codon usage of the tissue-specific transcriptome. Cancer disrupts the gene expression pattern of healthy tissue resulting in altered codon usage preferences. The topic of codon usage changes as they relate to codon demand, and tRNA supply in cancer is of growing interest.

**Methods:**

We analyzed transcriptome-weighted codon and codon pair usage based on The Cancer Genome Atlas (TCGA) RNA-seq data from 6427 solid tumor samples and 632 normal tissue samples. This dataset represents 32 cancer types affecting 11 distinct tissues. Our analysis focused on tissues that give rise to multiple solid tumor types and cancer types that are present in multiple tissues.

**Results:**

We identified distinct patterns of synonymous codon usage changes for different cancer types affecting the same tissue. For example, a substantial increase in GGT-glycine was observed in invasive ductal carcinoma (IDC), invasive lobular carcinoma (ILC), and mixed invasive ductal and lobular carcinoma (IDLC) of the breast. Change in synonymous codon preference favoring GGT correlated with change in synonymous codon preference against GGC in IDC and IDLC, but not in ILC. Furthermore, we examined the codon usage changes between paired healthy/tumor tissue from the same patient. Using clinical data from TCGA, we conducted a survival analysis of patients based on the degree of change between healthy and tumor-specific codon usage, revealing an association between larger changes and increased mortality. We have also created a database that contains cancer-specific codon and codon pair usage data for cancer types derived from TCGA, which represents a comprehensive tool for codon-usage-oriented cancer research.

**Conclusions:**

Based on data from TCGA, we have highlighted tumor type-specific signatures of codon and codon pair usage. Paired data revealed variable changes to codon usage patterns, which must be considered when designing personalized cancer treatments. The associated database, CancerCoCoPUTs, represents a comprehensive resource for codon and codon pair usage in cancer and is available at https://dnahive.fda.gov/review/cancercocoputs/. These findings are important to understand the relationship between tRNA supply and codon demand in cancer states and could help guide the development of new cancer therapeutics.

**Supplementary Information:**

The online version contains supplementary material available at 10.1186/s13073-021-00935-6.

## Background

While our understanding of the genetic basis for various cancer types has improved markedly in recent years, much remains to be explored and elucidated. Since 2006, with the advent of next-generation sequencing, RNA-seq has been leveraged to investigate the transcriptome of cancer cells [1]. Shortly thereafter, The Cancer Genome Atlas (TCGA) of the National Cancer Institute, National Institutes of Health, released its first publication investigating the gene expression in human glioblastomas [2]. Furthermore, genetic sequencing has identified many somatic mutations that are predictive of cancer development, progression, and the alteration of downstream pathways [3]. A multitude of studies have focused on specific mutations and their impact on the cancer phenotype, particularly the tumor suppressor gene p53 and oncogenes of the Ras family [4–7]. Other noteworthy cancer-related genes that have been extensively studied include breast cancer type 1 susceptibility protein (*BRCA1*) and breast cancer type 2 susceptibility protein (*BRCA2*), adenomatous polyposis coli (*APC*), and epidermal growth factor receptor (*EGFR*) in various types of breast, colorectal, and lung cancer, respectively [8–10].

While cancer-associated somatic mutations are often missense, deletions, or insertions, it has been estimated that synonymous single nucleotide polymorphisms (SNPs) that do not affect the amino acid sequence of a gene account for ~ 6–8% of cancer driver mutations. These mutations are frequently associated with transcript splicing dysregulation [11]. Furthermore, synonymous mutations have been shown to affect the expression and mRNA stability of the KRAS proto-oncogene (*KRAS*) [12, 13] and the synonymous codon usage bias of the *KRAS* gene itself is associated with enhanced translation efficiency during cell proliferation [14]. Still, other studies have highlighted synonymous cancer driver mutations that are unrelated to disruptions in splicing [13, 15]. One group found that a synonymous mutation in *Tristetraprolin* was associated with a lack of response to Herceptin in human epidermal growth factor receptor 2 (*HER2*) type breast cancer patients due to decreased translation efficiency of the gene [16]. In a recent study, Teng et al. described elevated ratios of post-transcriptionally impaired synonymous variants associated with 22 cancer types and, notably, poor prognosis for 5 of those cancer types [17]. This body of evidence underscores the necessity to further unravel the relationship between changes in synonymous codon usage, their cognate tRNA abundance, cellular growth state, and cancer progression.

Although many studies have concentrated on specific mutations in tumor-associated genes, less focus has been given to global changes in codon usage within cancerous tissue. The redundancy in the genetic code gives rise to codon usage bias, a phenomenon affecting all domains of life wherein synonymous codons are differentially utilized within an organism’s transcriptome [18, 19]. This observation also applies to two consecutive codons, termed a codon pair, with the frequency of codon pairs occurring in a non-random fashion that is not predictable from codon usage frequencies alone [20, 21]. In multi-cellular organisms, this phenomenon extends to codon usage within a particular tissue, whereby the differential gene expression profile of a tissue dictates its codon and codon pair usage [22, 23]. This phenomenon is relevant to the design of tissue-specific gene therapies, and in the case of cancer, may be useful in the design of recombinant mRNA-based cancer vaccines [24].

An important aspect of codon usage is its interplay with the local tRNA repertoire. The correlation between codon usage frequencies and cognate tRNA abundance has long been established in *Escherichia coli* and *Saccharomyces cerevisiae* [25, 26]. This correlation is also observed dynamically in *Escherichia coli* as the codon usage frequencies of the transcriptome and tRNA repertoire change during different growth phases [27]. This phenomenon is associated with the faster translation of more frequently used codons [28]. While this direct relationship has been more difficult to establish in multi-cellular organisms, some important studies have investigated the relationship between tissue-specific codon usage and tRNA expression in *Drosophila melanogaster* and *Homo sapiens* [29, 30]. A study by Dittmar et al. reported human tissue-specific changes in tRNA species, using a tRNA-specific microarray [30]. They also found a significant correlation between liver codon usage and cognate tRNA expression, which they postulated could be explained by the codon usage of highly expressed liver-specific genes [30]. Additionally, a study by Gingold et al. found that the cognate tRNA pool closely matches the codon usage signatures of proliferative and differentiated human cells, highlighting the potential relationship between tRNA supply and codon demand in cancerous cells and tissue [31]. However, to fully understand the implications of changes to the tRNA pool, it is essential to characterize the dynamic codon usage landscape between differentiated, proliferative, and cancerous cells.

Indeed, recent studies have explored the impact of codon usage on cancer. A noteworthy study by Hernandez-Alias et al. found intriguing changes in translation efficiency of synonymous codons for arginine and threonine. Namely, they described consistent, significant preference for Arg-AGA in 15 analyzed cancer types and preference for Thr-ACG in 12 of 16 cancer types [32]. They also highlighted a strong association between preferential Arg-AGA usage and poor cancer prognosis. Another investigation by Bin et al. identified synonymous mutation hotspots in tumor samples from TCGA. They compared the signatures of these mutations with those of the 1000 Genomes Project, highlighting a preference for synonymous G:C - > A:T transitions in TCGA compared to T:A - > C:G in 1000 Genomes, which resulted in AT enrichment of synonymous codons in cancer samples [33]. Furthermore, an intriguing recent publication found that proliferation-associated transcripts were enriched in rare codons and that their increased translation efficiency was not associated with changes in tRNA abundance between proliferative and non-proliferative states. While rare codons may be associated with translational bottlenecks in slowly dividing cells, the authors propose that this barrier is removed during proliferation, allowing for faster translation [34]. This is a noteworthy finding as an abundance of literature has focused on changes to the tRNA pool in proliferation and cancer and their associated impact on translation efficiency of cognate codons, while the previously mentioned study proposes an exclusively codon usage-based mechanism for this phenomenon. Regardless of the role of tRNA abundance, which remains under investigation, these findings have important implications for the role of synonymous mutations in cancer development and progression.

In the present study, we have leveraged the public datasets from TCGA [2] to investigate preferential codon and codon pair usage changes between the transcriptomes of cancer and normal tissues. While previously mentioned studies of the TCGA dataset have focused on the supply to demand adaptation of codons and synonymous mutation signatures [32, 33], our work focuses on the changes to global codon usage patterns between normal tissues and tumors. We utilized gene expression data from 6427 solid tumor samples and 632 normal tissue samples representing 32 cancer types affecting 11 human tissues. We have highlighted the findings in cancer types of diverse tissues, including liver, lung, breast, and prostate. We have also detailed codon and codon pair usage changes observed between paired normal and cancer tissue samples from individual patients, and conducted a survival analysis of patients with varying degrees of change between their healthy and tumor-specific codon and codon pair usage. Furthermore, we have created a database containing the codon and codon pair usage metrics of these 32 cancer types, which allows for comparison to normal tissue where available. The database can be accessed online at https://dnahive.fda.gov/review/cancercocoputs/ [35] and provides the user with a choice of heatmap visualization for codon pair usage metrics, including frequency per million codon pairs, percentile rank, and observed/expected ratio. The analysis presented herein adds to the understanding of global codon usage patterns associated with malignant neoplasia and has implications for cancer treatment strategies. This applies specifically to the design of personalized cancer vaccines, where the codon usage landscape of an individual patient may dictate the design of mRNA-based therapies. The associated database represents the most comprehensive source for cancer-specific codon and codon pair usage information to date.

## Methods

### Data acquisition and sample selection

RNA-seq files were downloaded from the National Cancer Institute’s Genomic Data Commons (GDC) repository [36]. As of 07/30/2020 (date of download), there were 16,175 RNA-seq, “HTSeq–Counts” files available under open access. Supplemental metadata files were downloaded from the GDC repository along with RNA-seq files. Each downloaded file had been assigned a file ID (a unique identifier for each RNA-seq file). Metadata files connected case ID (a unique identifier for each patient) with all file IDs associated with that patient.

Only “primary tumor” and “solid tissue normal” samples were used, which excluded 3644 samples. Only tissue samples from patients who had not received prior treatment were used, resulting in the exclusion of 1935 additional files. We focused on well-described solid tumors for which at least 3 normal tissue samples were available. We omitted hematologic tumors given the heterogeneous nature of the underlying cancers, choosing instead to focus on tissues that produce a limited number of tumor types from simple epithelia. This resulted in the exclusion of 3537 tissue samples from other organs and tissues. For the present study, data from 7059 tissue samples including 6427 primary tumor samples and 632 normal tissue samples were used. Transcriptomic data from these samples constitutes TCGA data. We identified 600 pairs of matched normal tumor tissue data present within this data set and used these for paired tissue analysis. Paired analysis was performed on 29 out of 32 cancer types. Paired analysis was not applied to papillary transitional cell carcinoma (TCC) of the bladder, tubular adenocarcinoma of the stomach, and esophageal squamous cell carcinoma (SCC) as fewer than 3 patients with these diseases were identified in our data set. A more detailed description of samples included in each cancer type or normal tissue type can be found in Additional File 1: Table S1.

### Normal tissue type definition

We defined a primary normal tissue sample’s type based on one parameter: organ/tissue of origin. Samples with the same tissue of origin were grouped together. Specific groups of normal tissue were merged together where appropriate (for example “upper lobe, lung” and “lung not otherwise specified” were merged into a single “normal lung” tissue type).

### Cancer type definition

We defined a primary tumor sample’s type based on 2 parameters: organ/tissue of origin and primary diagnosis. Samples with the same diagnosis and tissue of origin were grouped together and some cancer types were merged together where appropriate (for example, “esophageal SCC, not otherwise specified” and “esophageal SCC, keratinizing, not otherwise specified” were merged into a single “esophageal SCC” tissue type). From these cancer types, we selected those with at least 3 tumor samples, homogenous tissue of origin, and suitable normal tissue type. For example, thyroid cancers were excluded due to the heterogenous nature of thyroid tissue, and brain cancers were excluded due to the absence of normal brain tissue.

### Transcriptome weighted codon usage and codon pair usage calculations

Codon and codon pair counts were prepared as two matrices where each value represents the number of times a particular codon or codon pair appears in the coding sequence (CDS) of a specific gene’s primary transcript. Multiplying a vector describing transcripts per million (TPM) for each gene by this matrix and normalizing the resulting vector yields transcriptome weighted codon usage or codon pair usage values.

For aggregate analysis, a median sample is constructed by computing the median TPM across all tissue samples for a particular normal or tumor tissue type. Codon and codon pair usage is subsequently calculated for the median sample from each normal or tumor tissue type and the gene-level codon and codon pair counts derived from *Homo sapiens* assembly GRCh38.p13 and Gencode V34 annotations. This calculation was applied to 32 cancer types and 14 normal tissue types. As in our previous database, Kames et al., we normalized codon usage vectors to one thousand and codon pair usage to one million [23]. Because TPM was calculated by aligning RNA-seq reads with a reference genome, the resulting codon and codon pair usage values do not account for sequence variation between samples. For paired analysis, this calculation was applied to each tissue sample separately. Codon usage was compared between a normal sample and a primary tumor sample labeled with identical case IDs.

### Statistics and analysis

All statistical tests were performed using Pandas [37] and SciPy [38] libraries using Python version 3.7 [39]. SciKit-learn [40] was used to compute mean squared error (MSE) and was used for principal component analysis (PCA). Lifelines [41] was used for Kaplan-Meier analysis. For the Wald tests, a Bonferroni correction factor of 3 was applied because 3 synonymous codons were tested. The null hypothesis that the slope is zero was rejected when a resulting *p*-value was less than $\frac{0.01}{3}$ (3.3E−3). For the Wilcoxon signed-rank tests, a Bonferroni correction factor of 64 was applied because 64 codons were tested. The null hypothesis that paired samples follow the same codon usage distribution was rejected when a resulting *p*-value was less than $\frac{0.01}{64}$ (1.6E−4).

### Figure preparation

Figures were prepared using the matplotlib [42] library along with Python version 3.7 [39]. The SciPy [38] library was used to create dendrograms illustrating Euclidean distance-based hierarchal clustering. The lifelines [41] package was used to plot Kaplan-Meier curves.

## Results

### Codon and codon pair usage patterns in normal and primary tumor tissues

We obtained RNA-seq data from TCGA [36] and sorted files into tissue groups based on sample type, diagnosis, and tissue of origin. For each tissue group, we computed median transcriptomic profiles and used these profiles to calculate median codon and codon pair usage for each tissue. We examined codon and codon pair usage for each tissue to better understand the relationships between cancer types and their respective normal tissues. A more detailed description of how tissue samples were assigned to each normal or cancer tissue type can be found in the “Methods” section and in Additional File 1: Table S1.

**Fig. 1:**
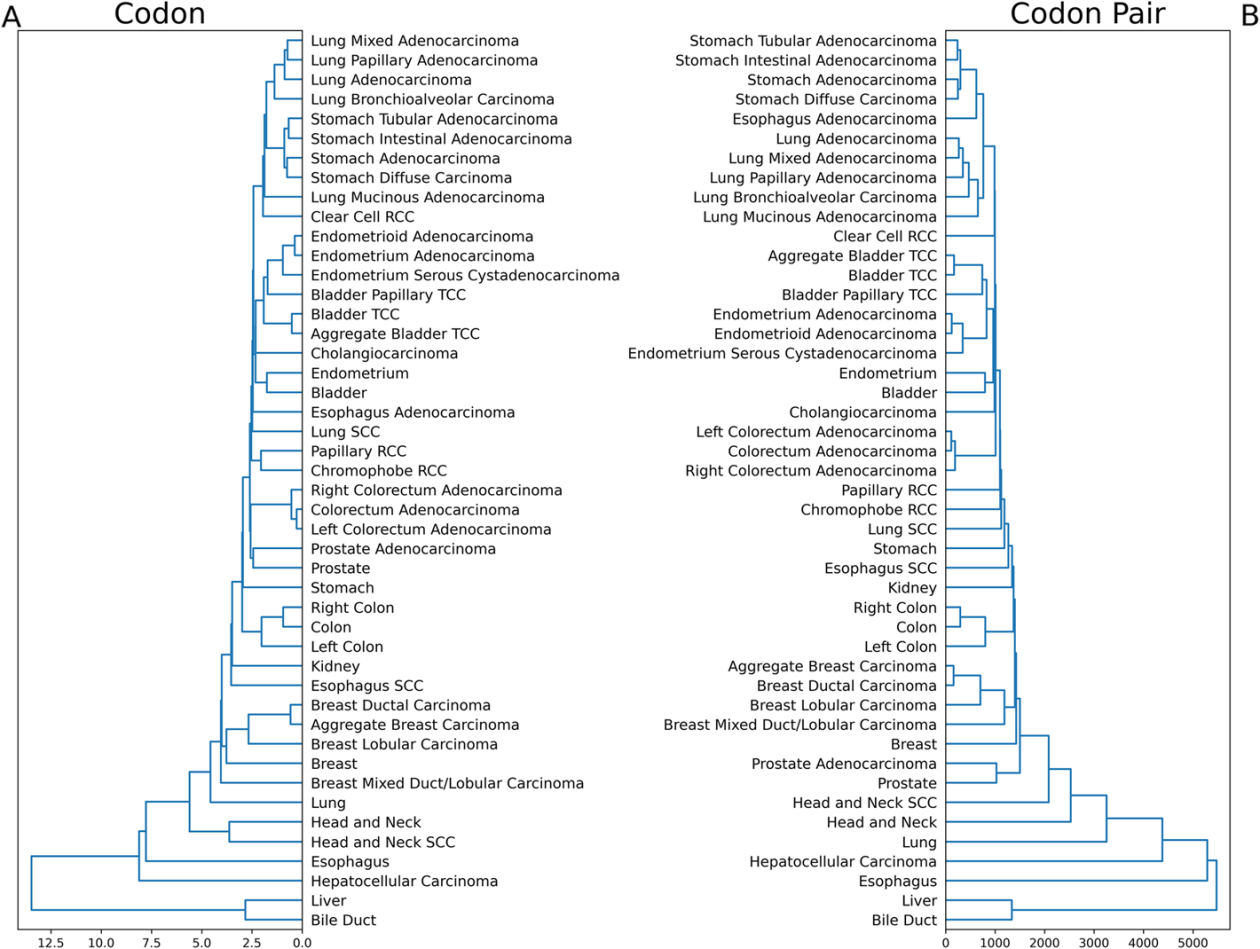
Primary tumor and normal tissue-specific codon and codon pair usage. **A**, **B** Euclidean distance dendrogram which clusters tissues based on codon usage (**A**) or codon pair usage (**B**). Distance between tissues is reflected by the height of the parent node. Codon and codon pair usage values reflect the median tissue values for each primary tumor and normal tissue type

When tissues were clustered by Euclidean distance computed based on their codon usage (Fig. 1A) or codon pair usage (Fig. 1B), we observed similar clustering patterns. For example, 4 subtypes of lung adenocarcinoma (LUAD) are more similar to each other than they are to normal lung tissue or to lung squamous cell carcinoma (LUSC) according to both dendrograms; normal bladder tissue is the most similar to normal endometrial tissue than to any other tissue, and transitional cell carcinoma of the bladder is most similar to endometrial adenocarcinomas than other tissues; normal liver and normal bile duct tissue are more similar to each other, but hepatocellular carcinoma and cholangiocarcinoma do not cluster together.

Clustering of tissues can be attributed to similarity in patterns of codon usage (Additional File 2: Figs. S1 and S2). GAG, CTG, and AAG are highly used while TCG is rarely used across all tissue types. As expected, stop codons TGA, TAA, and TAG are consistently the most rarely used codons. Normal liver and bile duct have strikingly similar patterns of codon usage which differ from hepatocellular carcinomas and cholangiocarcinomas, respectively. For example, GAG and AAA usage is higher in normal liver and bile duct tissue than in hepatocellular carcinoma and cholangiocarcinoma (Additional File 2: Fig. S1). In contrast, the difference in codon usage between normal prostate tissue and prostate adenocarcinoma is nearly imperceptible (Additional File 2: Fig. S1).

### Cancer type-specific changes in codon and codon pair usage

**Fig. 2:**
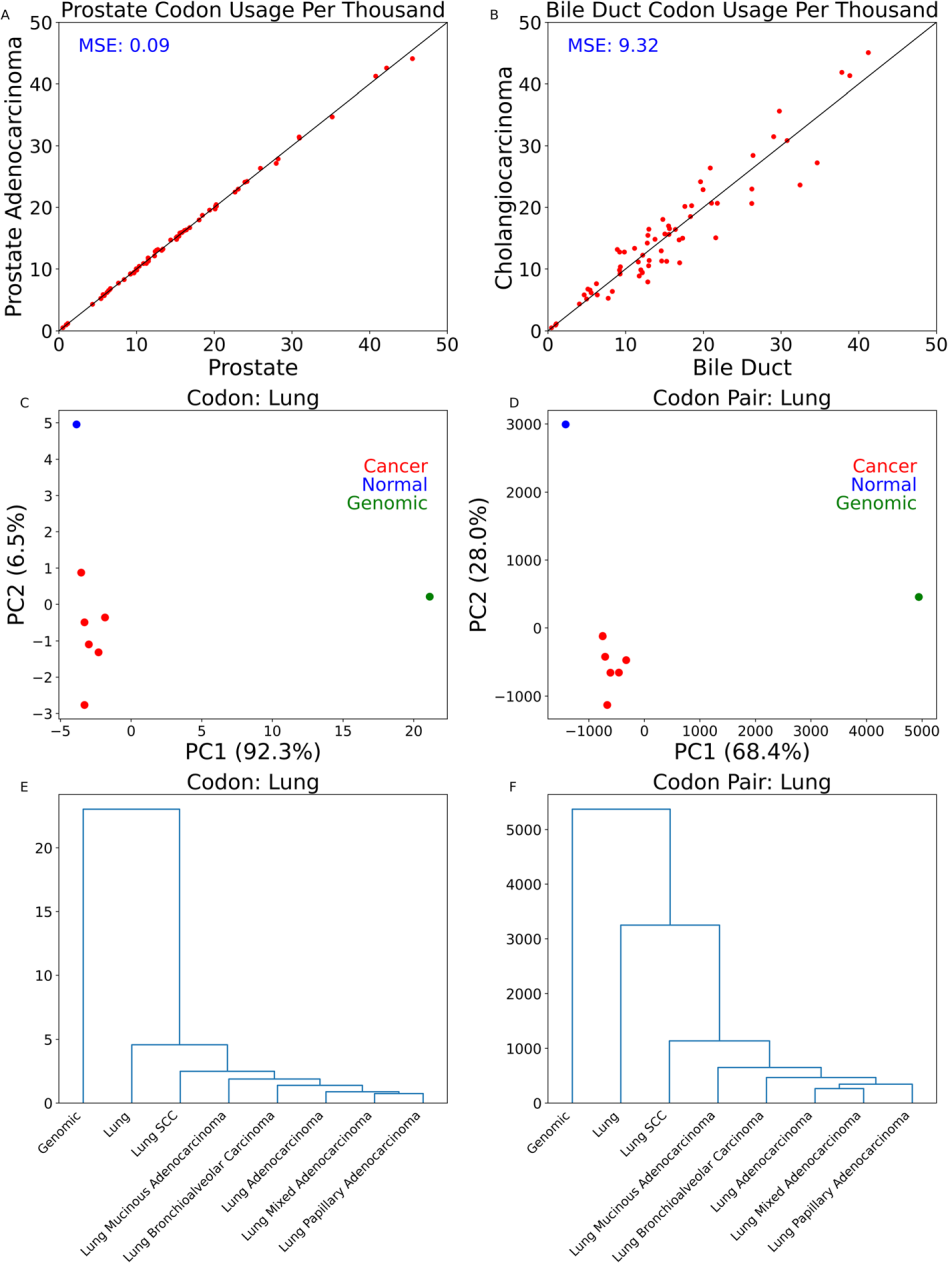
Aggregate normal vs. cancer codon and codon pair usage comparison for select tissues. **A**, **B** Scatter plots comparing codon usage between prostate adenocarcinoma and normal prostate tissue (**A**) and between cholangiocarcinoma and normal bile duct tissue (**B**). Each red point represents a codon. Codons above the black diagonal line are more frequent in cancer tissue than normal tissue. The mean square error (MSE) value is noted in the top left of the graph. A higher MSE value indicates more difference between codon usage in the primary tumor tissue and codon usage in normal tissue. **C**, **D** Principal component analysis for codon (**C**) and codon pair (**D**) usage in normal lung tissue, non-small cell lung cancer tissues, and genomics. Genomic codon and codon pair usage values are not transcriptome weighted. **E**, **F** Euclidean distance dendrograms based on tissue-specific codon usage (**E**) or codon pair usage (**F**)

If no codon usage difference existed between primary tumor tissue and its respective normal tissue, we would expect primary tumor codon usage to be equal to normal tissue codon usage. Based on this, we quantified the level of overall codon usage change by MSE for each of 32 cancer types. Prostate adenocarcinoma (Fig. 2A) showed relatively low MSE compared to other cancer types (0.09) indicating it is a cancer type with a relatively low difference in codon usage between tumor and normal tissue. In contrast, cholangiocarcinoma (Fig. 2B) showed the highest MSE of all cancer types (9.32) indicating it is the cancer type with the most difference in codon usage between tumor and normal tissue. The median MSE of all considered cancer types was 0.40 (Additional File 3: Table S2).

In order to characterize codon usage differences, for each cancer type, we identified 10 codons with the greatest difference in usage between primary tumor tissue and its respective normal tissue. Half of these codons have higher usage in cancer tissue than in normal tissue while the other half have higher usage in normal tissue (Table 1; Additional File 4: Table S3). As expected, larger changes in individual codon’s usage were observed in cancer types with higher MSE. For example, CGG-Arg usage was more than 47% higher in cholangiocarcinoma than in normal bile duct tissue, and TGT-Cys usage was more than 61% higher in normal bile duct than in cholangiocarcinoma. In contrast, the codons with the most differences in usage between prostate adenocarcinoma and normal prostate were GGT-Gly (less than 4% higher in prostate adenocarcinoma) and ATA-Ile (less than 4% higher in normal prostate).

**Table 1:** Codon usage differences between each cancer and its respective normal tissue

Cancer name	Higher in cancer or normal	Codon	% difference
Aggregate transitional cell carcinoma—bladder	Cancer	CGG	5.42
Aggregate transitional cell carcinoma—Bladder	Normal	TGT	5.23
Transitional cell carcinoma—bladder	Cancer	CGG	4.76
Transitional cell carcinoma—bladder	Normal	TGT	5.03
Papillary transitional cell carcinoma—bladder	Cancer	CGG	8.26
Papillary transitional cell carcinoma—bladder	Normal	CCT	9.28
Aggregate carcinoma—breast	Cancer	GGT	15.83
Aggregate carcinoma—breast	Normal	TGT	7.44
Ductal carcinoma—breast	Cancer	GGT	15.06
Ductal carcinoma—breast	Normal	TGT	7.60
Lobular carcinoma—breast	Cancer	GGT	19.60
Lobular carcinoma—breast	Normal	TTA	8.93
Duct and lobular carcinoma—breast	Cancer	GGT	28.12
Duct and lobular carcinoma—breast	Normal	TGT	5.01
Colorectal adenocarcinoma	Cancer	CGT	11.28
Colorectal adenocarcinoma	Normal	TGC	8.23
Left colorectal adenocarcinoma	Cancer	CGT	11.39
Left colorectal adenocarcinoma	Normal	TGC	7.55
Right colorectal adenocarcinoma	Cancer	CGT	12.23
Right colorectal adenocarcinoma	Normal	TGC	10.40
Adenocarcinoma—endometrium	Cancer	GCG	6.06
Adenocarcinoma—endometrium	Normal	CAA	8.52
Endometrioid adenocarcinoma	Cancer	GCG	6.07
Endometrioid adenocarcinoma	Normal	CAA	9.01
Serous cystadenocarcinoma—endometrium	Cancer	GCG	6.86
Serous cystadenocarcinoma—endometrium	Normal	CAA	7.22
Squamous cell carcinoma—head and neck	Cancer	TCC	6.20
Squamous cell carcinoma—head and neck	Normal	CAC	4.78
Squamous cell carcinoma—esophagus	Cancer	CGC	19.16
Squamous cell carcinoma—esophagus	Normal	TAT	22.41
Esophageal adenocarcinoma	Cancer	CGG	17.63
Esophageal adenocarcinoma	Normal	TAT	18.16
Clear cell renal cell carcinoma	Cancer	CGC	6.67
Clear cell renal cell carcinoma	Normal	ATA	6.57
Papillary renal cell carcinoma	Cancer	CGC	11.36
Papillary renal cell carcinoma	Normal	TTA	13.01
Chromophobe renal cell carcinoma	Cancer	CGC	10.11
Chromophobe renal cell carcinoma	Normal	TGC	10.03
Hepatocellular carcinoma	Cancer	CGG	24.19
Hepatocellular carcinoma	Normal	TGT	28.55
Cholangiocarcinoma	Cancer	CGG	47.48
Cholangiocarcinoma	Normal	TGT	61.40
Adenocarcinoma—lung	Cancer	CGT	11.77
Adenocarcinoma—lung	Normal	TGC	13.07
Squamous cell carcinoma—lung	Cancer	CGT	16.52
Squamous cell carcinoma—lung	Normal	TGC	17.95
Adenocarcinoma with mixed subtypes—lung	Cancer	CGT	11.86
Adenocarcinoma with mixed subtypes—lung	Normal	TGT	12.38
Bronchioloalveolar carcinoma	Cancer	TTA	12.15
Bronchioloalveolar carcinoma	Normal	TGC	10.07
Papillary adenocarcinoma—lung	Cancer	CGT	10.82
Papillary adenocarcinoma—lung	Normal	TGT	11.76
Mucinous adenocarcinoma—lung	Cancer	CGG	8.76
Mucinous adenocarcinoma—lung	Normal	CCT	8.90
Prostate adenocarcinoma	Cancer	GGT	3.74
Prostate adenocarcinoma	Normal	ATA	3.57
Adenocarcinoma—stomach	Cancer	TTA	12.74
Adenocarcinoma—stomach	Normal	TGC	5.62
Intestinal type adenocarcinoma—stomach	Cancer	TTA	12.45
Intestinal type adenocarcinoma—stomach	Normal	TGC	5.82
Diffuse type carcinoma—stomach	Cancer	TTA	13.49
Diffuse type carcinoma—stomach	Normal	GTC	5.68
Tubular adenocarcinoma—stomach	Cancer	TTA	13.51
Tubular adenocarcinoma—stomach	Normal	TGC	6.88

This table describes the most pronounced codon usage differences for each cancer type based on median transcriptome-weighted codon usage comparison between each cancer type and its respective normal tissue type. For each cancer type, one codon with higher usage in primary tumor tissue and one codon with higher usage in normal tissue are listed. More codon differences can be found in Additional File 4: Table S3

To better contextualize these codon usage differences, we next examined the differences between cancer and normal tissues’ codon and codon pair usages relative to non-transcriptomic weighted (“genomic”) codon and codon pair usage. By PCA of codon usage (Fig. 2C) and of codon pair usage (Fig. 2D), we observe the clear separation of lung tissues from genomic samples and separation of normal lung tissue from primary lung tumors. We also compared codon and codon pair usage differences between lung tissues by hierarchal clustering. Clustering patterns are similar between codon usage (Fig. 2E) and codon pair usage (Fig. 2F). In both, LUAD subtypes (mixed adenocarcinoma, papillary adenocarcinoma, bronchoalveolar carcinoma, mucinous adenocarcinoma) are more similar to each other than they are to LUSC or to normal lung tissue. However, there appears to be relatively more difference between normal lung tissue and primary non-small cell lung cancer tissues when looking at the codon pair usage than at the codon usage.

Similar to our analysis comparing lung cancer types with normal lung tissue, we also examined breast, endometrial, esophageal, and gastric cancer types in relation to their respective normal tissue and to non-transcriptomic-weighted (genomic) codon and codon pair usage. We observed similar clustering patterns when examining breast, endometrial, esophageal, gastric, bladder, and kidney tissues (Additional File 2: Fig. S3). Notably, codon pair usage for esophageal adenocarcinoma and esophageal SCC is more similar to non-transcriptomic weighted (genomic) codon pair usage than to normal esophageal codon pair usage.

### Aggregate comparison of different cancers originating from similar organs and tissues

**Fig. 3:**
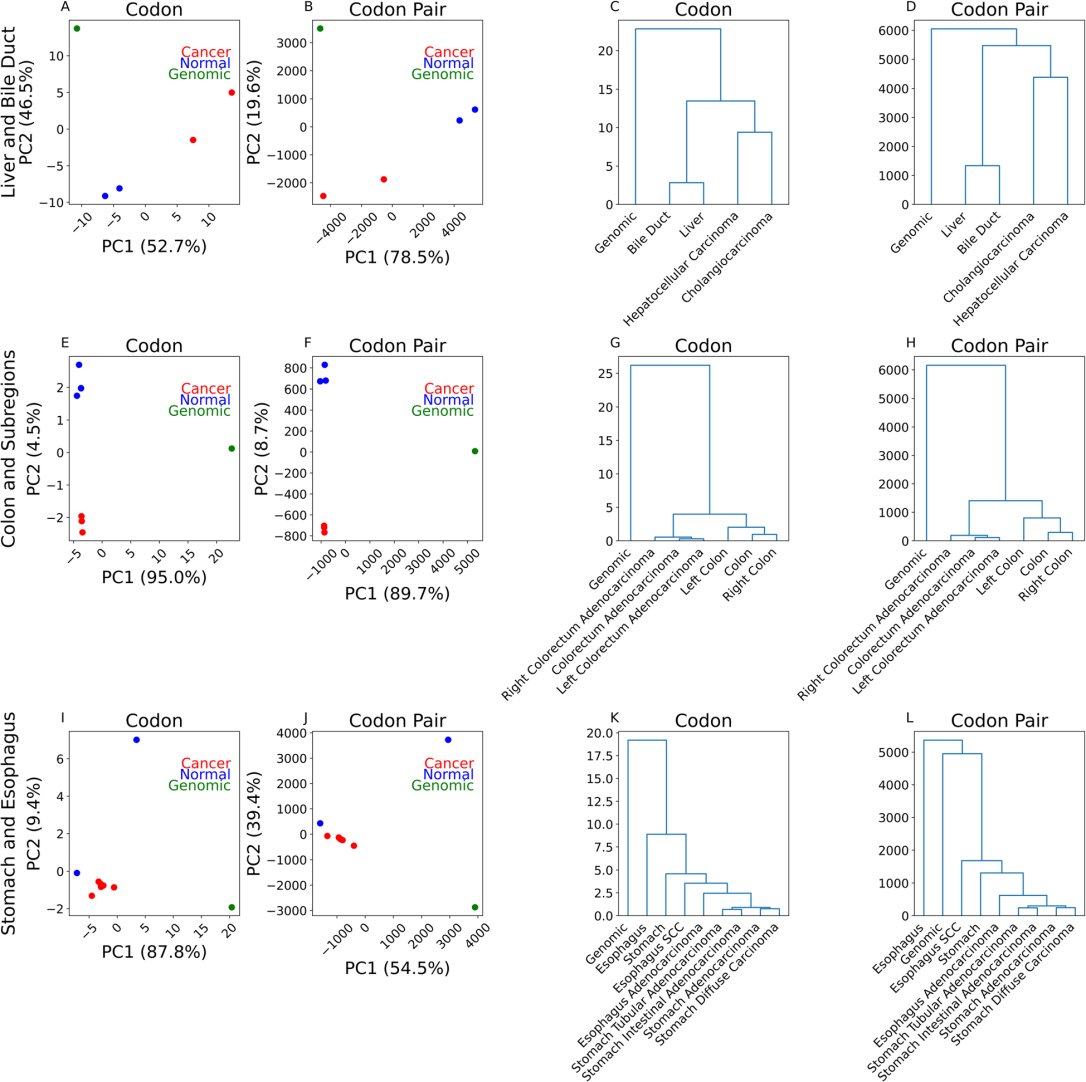
Aggregate comparison for select primary tumor types. **A**, **B** Principal component analysis for liver and bile duct tissues based on codon usage (**A**) and codon pair usage (**B**). **C**, **D** Euclidean distance dendrogram for liver and bile duct tissues based on codon usage (**C**) and codon pair usage (**D**). **E**, **F** Principal component analysis for colorectal tissues based on codon usage (**E**) and codon pair usage (**F**). “Right” colon refers to the ascending colon, cecum, and hepatic flexure of the colon. “Left” colon refers to the descending colon, splenic flexure of the colon, sigmoid colon, rectosigmoid junction, and rectum. **G**, **H** Euclidean distance dendrograms for colorectal tissues based on codon usage (**G**) and codon pair usage (**H**). **I**, **J** Principal component analysis for gastric and esophageal tissues based on codon (**I**) and codon pair usage (**J**). **K**, **L** Euclidean distance dendrograms for gastric and esophageal tissues based on codon (**K**) and codon pair usage (**L**)

In a previous study, we demonstrated tissue-specific codon and codon pair usage signatures [23]. After comparing cancer tissues with their respective normal tissue, we next examined the differences in codon and codon pair usage signatures between cancer types. By PCA and hierarchal clustering, we found that cholangiocarcinoma and hepatocellular carcinoma were more similar to each other than their respective normal tissues based on codon usage (Figs. 3A, C) and codon pair usage (Figs. 3B, D). Interestingly, a higher level of difference is seen between cholangiocarcinoma and hepatocellular carcinoma than between their respective normal tissue types.

We next examined primary cancer tissues arising from the colon and its subregions. By PCA based on codon usage (Fig. 3E) and codon pair usage (Fig. 3F), we see the separation of normal colorectal tissue from colorectal adenocarcinoma purely along PC2. PC2 accounted for 4.2% of the variation in codon usage data and 8.5% of the variation in codon pair usage data, implying that the difference between normal colorectal tissues and colorectal adenocarcinoma is relatively small compared to the difference between their codon and codon pair usage and non-transcriptomic-weighted (genomic) codon and codon pair usage. By hierarchal clustering, we also see a low difference in codon usage (Fig. 3G) and codon pair usage (Fig. 3H) between colorectal tissues with genomic as a clear out-group.

Following these findings, we considered cancers arising from the esophagus and stomach. By PCA, we observed that primary gastric and esophageal adenocarcinomas are more similar to each other and normal stomach than they are to normal esophagus based on codon usage (Fig. 3I) and codon pair usage (Fig. 3J). By hierarchal clustering based on codon usage (Fig. 3K) and codon pair usage (Fig. 3L), we see gastric cancers (tubular adenocarcinoma, intestinal adenocarcinoma, diffuse carcinoma, and adenocarcinoma) are more similar to each other than to normal stomach tissue or any esophageal tissues. We see esophageal adenocarcinoma is more similar to gastric cancers than to esophageal SCC or normal esophageal tissue (Figs. 3K, L). While codon and codon pair usage-based clustering results are very similar, there is a notable difference. By codon pair clustering, esophageal cancer tissues are more similar to genomic (or non-transcriptomic weighted) codon pair usage than to normal esophageal codon pair usage (Fig. 3L). However, esophageal cancer tissues are more similar to normal esophagus codon usage than to genomic codon usage (Fig. 3K).

In our fourth set of comparisons, we analyzed SCC from the lung and SCC from the head and neck to see how codon and codon pair usage differed between similar cancer types arising from distinct tissues of origin. By PCA and hierarchal clustering based on codon usage, we observed that SCCs were more similar to their respective normal tissues than to each other (Additional File 2: Fig. S4A, C). However, based on codon pair usage, SCC tissues were more similar to each other than to their respective normal tissues (Additional File 2: Fig. S4B, D).

### Changes in codon usage accompany changes in codon preference

**Fig. 4:**
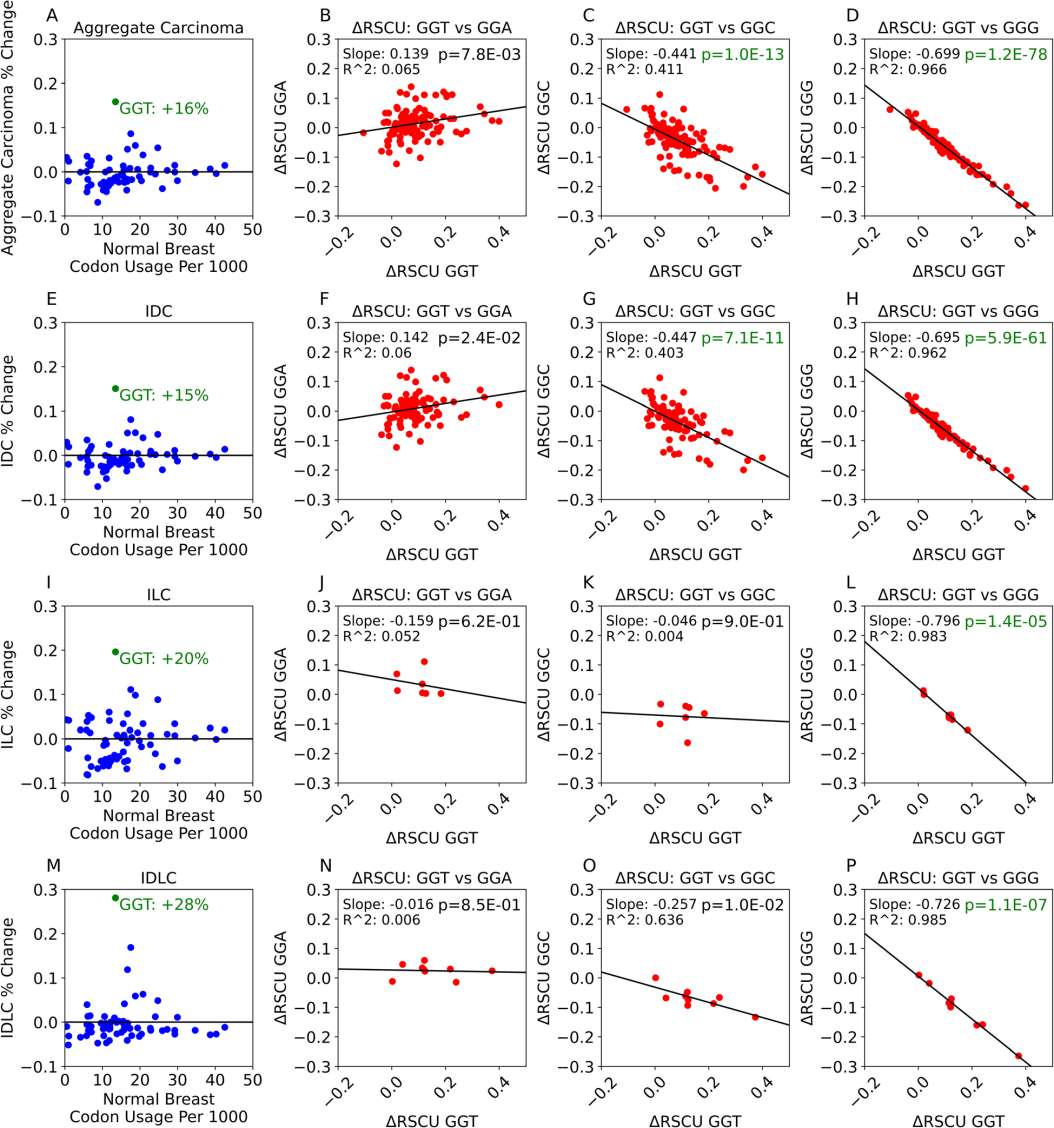
Change in codon usage and change in RSCU in breast cancers. **A** Scatterplot representing codon usage difference between normal breast tissue and aggregate breast cancer based on the median tissue values. Values along the *x*-axis represent the codon usage per thousand in normal breast tissue. Values along the *y*-axis represent the percent difference between aggregate breast cancer and normal breast usage. **B**–**D** Scatterplots representing the correlation between change in relative synonymous codon usage (RSCU) of GGT and its synonymous codons GGA (**B**), GGC (**C**), and GGG (**D**). Each point represents a change in individual patients (*n* = 107). *p*-value text appears green where the null hypothesis may be rejected (see the “Methods” section for the explanation of the Wald test used). **E** Scatterplot representing the codon usage difference between normal breast tissue and invasive ductal carcinoma (IDC) of the breast based on the median tissue values. Values along the *x*-axis represent the codon usage per thousand in normal breast tissue. Values along the *y*-axis represent the percent difference between IDC and normal breast usage. **F**–**H** Scatterplots representing the correlation between change in relative synonymous codon usage (RSCU) of GGT and its synonymous codons GGA (**F**), GGC (**G**), and GGG (**H**). Each point represents a change in individual IDC patients (*n* = 85). *p*-value text appears green where the null hypothesis may be rejected (see the “Methods” section for the explanation of the Wald test used). **I** Scatterplot representing the codon usage difference between normal breast tissue and invasive lobular carcinoma (ILC) based on the median tissue values. **J**–**L** Scatterplots representing the correlation between change in RSCU of GGT and its synonymous codons GGA (**J**), GGC (**K**), and GGG (**L**). Each point represents a codon change in individual ILC patients (*n* = 7). *p*-value text appears green where the null hypothesis may be rejected (see the “Methods” section for the explanation of the Wald test used). **M** Scatterplots representing the codon usage difference between normal breast tissue and mixed invasive ductal and lobular carcinoma (IDLC) based on the median tissue values. **N**–**P** Scatterplots representing the correlation between change in RSCU of GGT and its synonymous codons GGA (**N**), GGC (**O**), and GGG (**P**). Each point represents the change in individual IDLC patients (*n* = 9). *p*-value text appears green where the null hypothesis may be rejected (see the “Methods” section for the explanation of the Wald test used)

Median codon usage, referring to codon usage computed based on median transcriptomic weights from all tissue samples of a particular primary tumor or normal tissue type, cannot be used to detect variation in codon usage changes between patients. Having examined median codon and codon pair usage, we next looked at codon usage changes in individual patients by comparing primary tumor samples with normal tissue samples collected from the same patient. Of particular interest was the impact changes in codon usage have on codon preference measured by relative synonymous codon usage (RSCU). Based on median codon usage, we found that GGT-Gly consistently showed the greatest change in codon usage between 4 breast cancer groupings (aggregate breast cancer, IDC, ILC, IDLC) and normal breast tissues (Figs. 4A, E, I, M) with IDLC showing the greatest increase in GGT-Gly (+ 28%, Fig. 4 M). By the Wilcoxon signed-rank test, we found a change in GGT usage to be significant in aggregate breast cancer and IDC (Additional File 5: Table S4, Additional File 6: Table S5).

We next compared the change in RSCU for GGT with a change in RSCU for other glycine encoding codons in the 4 breast cancer groupings. All groupings show a weak correlation between GGT and GGA (Fig. 4B, F, J, N), the strongest of which appears in the aggregate breast cancer group (Fig. 4B: *R*^2^ = 0.065, $\frac{\Delta  \mathrm{GGA}}{\Delta  \mathrm{GGT}}=+0.139$). Three of the four groupings (all but ILC) showed a moderate correlation between GGT and GGC (Fig. 4C, G, K, O), the strongest of which occurs in IDLC (Fig. 4O: *R*^2^ = 0.636, $\frac{\Delta  \mathrm{GGC}}{\Delta  \mathrm{GGT}}=-0.257$). However, all 4 groupings displayed a strong and negative correlation between GGT and GGG (Fig. 4D, H, L, P: all *R*^2^ > 0.95 and all $\frac{\Delta  \mathrm{GGG}}{\Delta  \mathrm{GGT}}$ < − 0.69). We performed a two-sided Wald test to evaluate the null hypothesis that the slope of each graph is 0. With a Bonferroni correction value of 3 and alpha level of significance of 0.01, *p* values < 0.0033 were considered significant. We observed a strong and significant relationship between ΔRSCU of GGT and ΔRSCU of GGG for all breast cancer types. We also see a weaker correlation between GGT and GGC in ILC patients than in IDC or IDLC patients. While we observed a significant relationship between GGT and GGC in the aggregate breast cancer patient group (*n* = 107; *p* = 1.0E−13) and in IDC patients (*n* = 85; *p* = 7.1E−11), we did not observe a significant relationship between GGT and GGC in ILC patients (*n* = 7; *p* = 0.90) nor in IDLC patients (*n* = 9; *p* = 0.01).

### Variation in codon usage is cancer type specific

We were interested in evaluating whether median transcriptomic weighted codon and codon pair usage is a fair reflection of patients’ codon and codon pair usage. We computed MSE based on codon usage for each patient with each of 29 cancer types with sufficient paired tissue samples and present a summary of the results in Table 2. As expected, prostate adenocarcinoma patients had the lowest MSE (median value 0.42) while cholangiocarcinoma patients had the highest MSE (median value 9.95). However, we observed remarkably high variation in MSE of prostate adenocarcinoma patients.

**Table 2:** Summary of MSE variation among patients with each cancer type

Cancer type	Mean	Min	25 percentile	Median	75 percentile	Max	Range	Number of patients
Prostate adenocarcinoma	3.88	0.04	0.24	0.42	1.21	56.14	56.10	50
Squamous cell carcinoma—head and neck	3.24	0.26	1.25	1.84	3.46	35.24	34.97	40
Hepatocellular carcinoma	4.21	0.40	1.41	2.96	6.26	19.59	19.19	49
Clear cell renal cell carcinoma	1.02	0.09	0.27	0.53	0.84	15.59	15.50	71
Esophageal adenocarcinoma	5.35	0.17	1.50	4.67	7.05	15.51	15.33	7
Aggregate carcinoma—breast	1.62	0.17	0.46	0.92	1.91	12.30	12.13	107
Ductal carcinoma—breast	1.60	0.17	0.46	0.86	1.89	12.30	12.13	85
Colorectal adenocarcinoma	1.74	0.18	0.42	0.76	1.44	11.76	11.59	46
Right colorectal adenocarcinoma	2.95	0.34	0.51	1.22	3.66	11.76	11.42	15
Duct and lobular carcinoma—breast	2.07	0.37	1.01	1.51	1.99	7.63	7.26	9
Adenocarcinoma—endometrium	1.07	0.18	0.40	0.55	1.20	7.43	7.25	23
Endometrioid adenocarcinoma	1.20	0.18	0.40	0.59	1.28	7.43	7.25	19
Cholangiocarcinoma	9.42	6.62	6.95	9.95	10.64	13.58	6.96	9
Adenocarcinoma—stomach	1.98	0.39	0.74	1.66	2.68	7.31	6.92	27
Papillary renal cell carcinoma	0.63	0.07	0.20	0.44	0.71	4.79	4.73	31
Chromophobe renal cell carcinoma	1.06	0.10	0.32	0.64	1.43	4.47	4.37	23
Aggregate transitional cell carcinoma—bladder	1.22	0.14	0.47	1.02	1.67	3.95	3.81	18
Transitional cell carcinoma—bladder	1.19	0.14	0.45	0.99	1.58	3.95	3.81	17
Adenocarcinoma—lung	1.22	0.18	0.60	1.01	1.77	3.54	3.36	51
Squamous cell carcinoma—lung	1.34	0.03	0.64	1.14	1.93	3.30	3.27	48
Bronchioloalveolar carcinoma	1.67	0.51	0.73	0.96	2.25	3.54	3.03	3
Left colorectal adenocarcinoma	0.83	0.18	0.43	0.72	1.04	2.64	2.46	18
Lobular carcinoma—breast	1.23	0.17	0.82	1.01	1.61	2.60	2.43	7
Intestinal type adenocarcinoma—stomach	1.13	0.39	0.46	0.69	1.31	2.81	2.41	5
Adenocarcinoma with mixed subtypes—lung	1.22	0.39	0.46	1.22	1.98	2.07	1.68	4
Diffuse type carcinoma—stomach	2.54	1.51	2.39	2.61	3.07	3.13	1.63	5
Papillary adenocarcinoma—lung	1.33	0.88	0.99	1.09	1.55	2.00	1.12	3
Mucinous adenocarcinoma—lung	0.78	0.23	0.71	0.94	1.01	1.04	0.81	4
Serous cystadenocarcinoma—endometrium	0.49	0.26	0.42	0.51	0.58	0.69	0.43	4

This table summarizes the MSE computed for each patient with each of 29 cancer types based on codon usage. Values presented here better describe the spread of MSE values for a cancer type. Columns include “Min,” “Max,” “Range,” “Median,” and “Mean.” The number of patients examined for each cancer type is described under the “Number of patients” column; 25% and 75% refers to the first and third quartile, respectively, and may not be useful for cancer types with low patient numbers

**Fig. 5:**
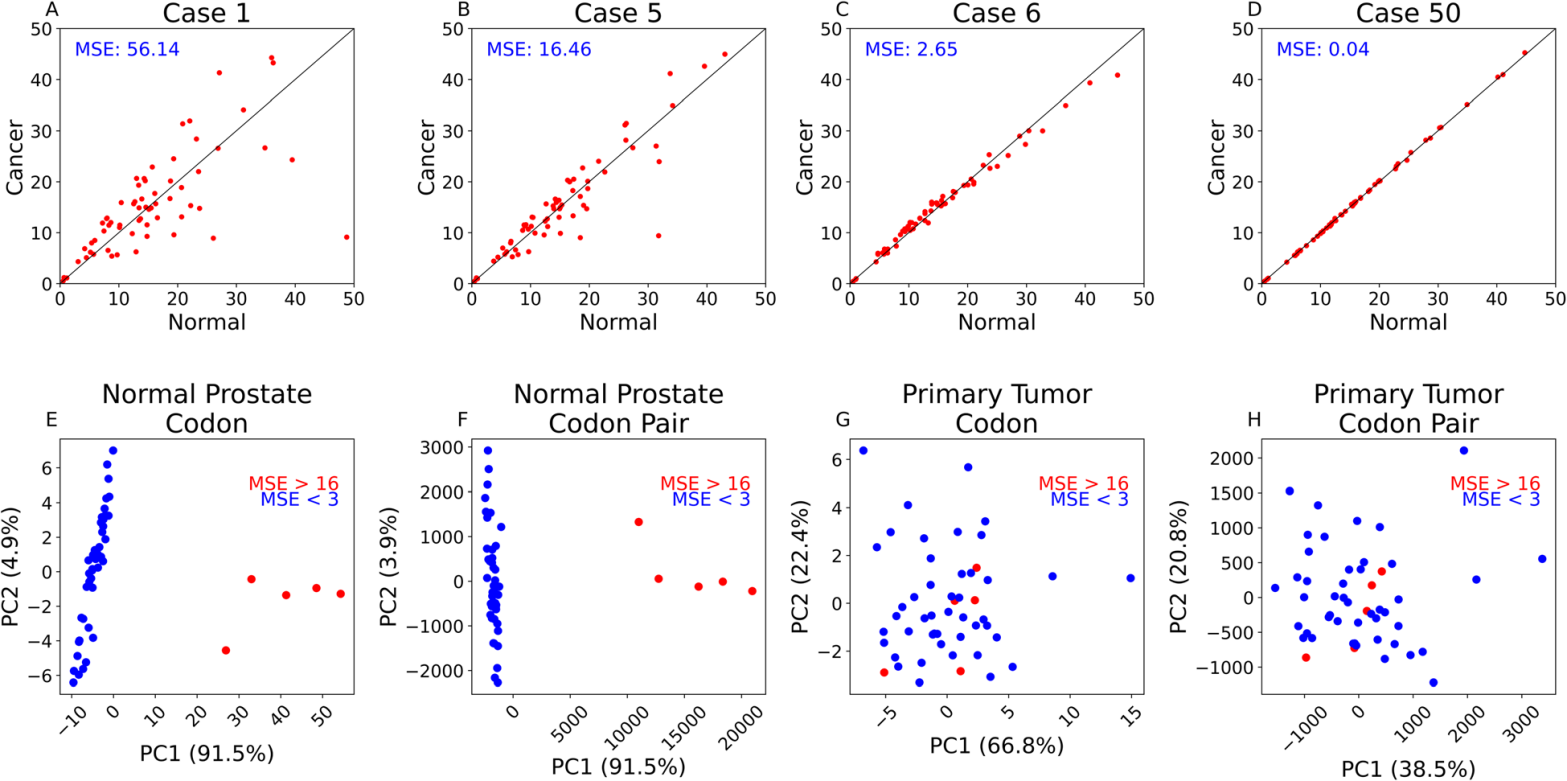
Codon and codon pair usage differences in prostate adenocarcinoma patients. **A**–**D** Representative scatterplots for 4 prostate adenocarcinoma patients. Case numbers are assigned in order of decreasing MSE (case 1 refers to the patient with the highest MSE while case 50 refers to the patient with the lowest MSE). **E**, **G** PCA based on codon usage in each patient’s normal prostate tissue (**E**) or primary tumor tissue (**G**). Cases with high MSE are colored red, and cases with low MSE are colored blue. **F**, **H** PCA based on codon pair usage in each patient’s normal prostate tissue (**F**) or primary tumor tissue (**H**). Cases with high MSE (> 16) are colored red, and cases with low MSE (< 3) are colored blue

To investigate this further, we assigned case numbers to patients based on MSE, so the patient with the highest MSE is referred to as “case 1” while the patient with the least MSE is referred to as “case 50.” Using the highest observed MSE from our median transcriptomic weighted codon usage analysis (cholangiocarcinoma, 9.32) as a threshold, we divided prostate adenocarcinoma patients into two groups: cases 1–5 (high MSE) and cases 6–50 (low MSE). MSE values observed in cases 1–5 ranged from 56.14 (Fig. 5A) to 16.46 (Fig. 5B). MSE values observed in cases 6–50 ranged from 2.65 (Fig. 5C) to 0.04 (Fig. 5D).

We next investigated the codon and codon pair usage differences between these two case groups. By PCA of both groups’ normal prostate samples, we see clear separation based on codon usage (Fig. 5E) and codon pair usage (Fig. 5F). However, we did not observe substantial differences in codon usage (Fig. 5G) or codon pair usage (Fig. 5H) when comparing primary tumor samples between these two groups of prostate adenocarcinoma cases.

### Global codon usage change predicts increased mortality

We next explored the impact of global codon and codon pair usage change on patient mortality. MSE was computed based on codon usage, codon pair usage, and raw transcriptomic weights (transcripts per million) using paired tumor and normal tissue samples collected from the same patient. A total of 596 patients with paired samples also had necessary clinical data for Kaplan-Meier analysis. These patients were divided into quartiles based on their MSE values, and the top quartile (25% of patients with highest MSE) and bottom quartile (25% of patients with lowest MSE) were compared. The probability of survival for each patient group was plotted over 10 years.

**Fig. 6:**
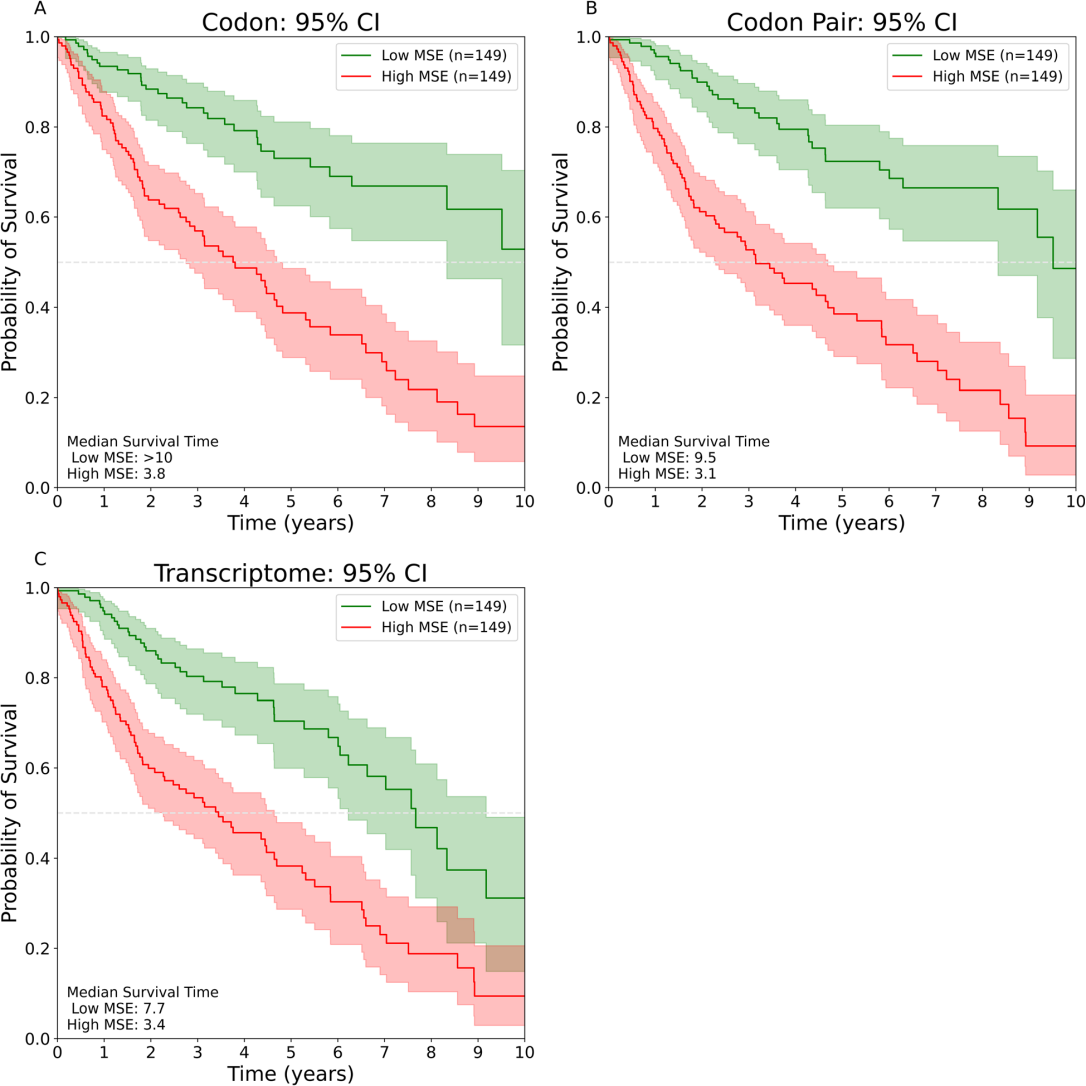
Kaplan-Meier analysis of codon, codon pair, and transcriptome changes in 596 patients. **A** Kaplan-Meier curves for patients with relatively high global codon usage changes (red) and patients with relatively low global codon usage changes (green). The horizontal dashed line represents 50% survival probability and intersects with each curve at their median survival time. Shaded regions represent 95% confidence intervals. **B** Kaplan-Meier curves for patients with relatively high global codon pair usage changes (red) and patients with relatively low global codon pair usage changes (green). The horizontal dashed line represents 50% survival probability and intersects with each curve at their median survival time. Shaded regions represent 95% confidence intervals. **C** Kaplan-Meier curves for patients with relatively high transcriptome changes (red) and patients with relatively low transcriptome changes (green). The horizontal dashed line represents 50% survival probability and intersects with each curve at their median survival time. Shaded regions represent 95% confidence intervals

We observed a clear separation between patients with high codon usage change and patients with low codon usage change (Fig. 6A). The median survival time of patients with high codon usage change was 3.8 years while the median survival time of the low codon usage change patient group was not reached after 10 years. After the first year, there was no overlap between the 95% confidence intervals for these two groups. We observed similar findings when patients were grouped according to codon pair usage changes (Fig. 6B). Patients with low codon pair usage changes had a median survival time of 9.5 years while patients with high codon pair usage changes had a median survival time of 3.1 years. Separating patients according to global transcriptomic change (Fig. 6C) also resulted in different mortality rates. The patient with low transcriptomic MSE had a median survival time of 7.7 years while patients with high transcriptomic MSE had a median survival time of 3.4 years. Unlike with codon and codon pair usage graphs, we observed less distinction between mortality rates of high MSE and low MSE patient groups when separating by transcripts per million as the 95% confidence intervals overlap after 8 years.

## Discussion

Synonymous codon usage bias is a phenomenon that has long been recognized in all domains of life and more recently across tissues of multi-cellular organisms [19, 20, 22, 23]. While synonymous mutations have historically been thought of as neutral and are often assumed to be benign, a critical mass of evidence suggests the opposite [43–45]. Examples include synonymous SNPs associated with both genetic disorders and cancer, due to a complex mechanism involving cognate tRNA abundance, mRNA stability and splicing, and translation kinetics [11, 46, 47]. While the process that leads from synonymous mutations to disease remains unsolved, it is imperative to document and describe associated changes in codon usage bias as well as divulge this information to the research community. In this study, we have described relevant changes in global codon and codon pair usage between the transcriptomes of healthy and cancerous tissue samples. The findings are based on genomic codon and codon pair counts weighted by gene expression derived from public data, and we have created an associated database (https://dnahive.fda.gov/review/cancercocoputs/) to access this information.

We began our investigation with an understanding that usage frequency and translation efficiency of synonymous codons are not necessarily equivalent. Previous work has highlighted substantial differences in transcriptomic-weighted codon and codon pair usage across 51 human tissues derived from the Genotype Tissue Expression (GTEx) Project [23, 48]. Varying degrees of bias were found measuring distances between the codon and codon pair usage of tissues and their effective numbers of codons and codon pairs. As biased codon usage has been established across healthy human tissues, we investigated its presence between normal and cancerous human tissues. Leveraging the publicly available RNA-seq data in TCGA, we first selected solid tumor types from the database for which there were also normal tissue data available (e.g., hepatocellular carcinoma and liver). We utilized the available transcriptomic data and transcript-level codon and codon pair usage to calculate distances between tissues and their respective cancers based on transcriptomic-weighted codon and codon pair usage. Both liver and bile duct stand out in their overall distance from their respective cancers (hepatocellular carcinoma and cholangiocarcinoma, respectively) and in specific codon usage differences between normal and tumor tissues. GAA and GAG are the only two synonymous codons for glutamic acid, while AAA and AAG are the only two for lysine. Both normal liver and bile duct tissues show little preference for either of these synonymous codons. However, hepatocellular carcinoma and cholangiocarcinoma show preference for GAG and AAG over GAA and AAA as evident by a shift in relative synonymous codon usage values away from 1.0 (Additional file 2: Fig S2). While more frequently used synonymous codons are associated with better translation efficiency, the increased usage of GAG and AAG in hepatocellular carcinoma and cholangiocarcinoma could also lead to additional translational pressure being placed on these codons. Such perturbations in synonymous codon usage can affect the protein structure through changes in co-translational folding kinetics [43, 49, 50]. Other changes to codon usage patterns between tissues and their respective cancers could have yet unknown impacts and warrant further investigation. Furthermore, while TCGA represents the most comprehensive single source for tumor-specific data, future studies could leverage additional repositories available in the International Cancer Genome Consortium Data Portal to validate and expand CancerCoCoPUTs [51].

To continue investigating changes in cancer-specific codon usage patterns, we examined other sets of normal and cancer tissue data from TCGA. Interestingly, not all tissues and respective tumor types were found to have such divergent codon usage patterns as seen with hepatobiliary cancers. For example, normal prostate and prostate adenocarcinoma have low MSE in normal vs tumor codon usage frequencies. This could be due to close similarity in gene expression profiles of normal prostate and prostate adenocarcinoma, which is often a slow-growing, less aggressive type of tumor. Investigation of the codon usage differences between proliferative and differentiated cells could shed light on this finding and would be an interesting topic for future research. On the other hand, the high MSE between the bile duct and cholangiocarcinoma is evidence of the dramatic changes that take place in codon usage between this tissue and its respective cancer.

We next examined different tumor types originating from the breast, lung, endometrium, and esophagus. Despite known differences between cancer types originating from the same tissue of origin (i.e., LUAD vs. LUSC), we found that codon usage and codon pair usage in these tumor types were generally more similar to each other’s codon and codon pair usage than they were to their respective normal tissue’s codon and codon pair usage, or to genomic codon and codon pair usage. These findings begged the question whether there is a relationship between synonymous codon preferences of cancers originating from the same tissue. We continued in this line of investigation by comparing changes in codon usage between the normal breast tissue and three breast cancer types (IDC, ILC, and IDLC). We found that GGT, the least frequent glycine codon in normal breast tissue (13.4 per 1000), was consistently the most elevated codon in IDC, ILC, and IDLC. We also found that increased RSCU of GGT consistently and strongly correlated with a decreased RSCU of GGG in all three cancer types compared to normal breast tissue. Similarly, increased RSCU of GGT correlated with a decreased RSCU of GGC, the most used glycine codon in normal breast tissue (24.7 per 1000 codons).

The over-representation of an otherwise rare codon (GGT) in these breast cancer types aligns with findings from Guimaraes et al., who recently observed elevated usage of rare codons in proliferating cells that led to enhanced translation efficiency [34]. The authors discussed the possibility that a bottleneck normally placed on the translation of rare codons in differentiated cells was alleviated during proliferation, allowing higher expression of proliferation-associated genes using rare codons [34]. It is noteworthy that their findings were observed in the absence of a significant change to the tRNA pool, suggesting that rare codon usage alone could drive increased translation efficiency in proliferative cells. However, it should also be noted that another recent study highlighted poor translational adaptation of other codons, such as AGA and ACT, in cancer based on the supply-to-demand ratio of tRNA abundance to codon usage [32, 52]. Although our findings are not contradictory, the clear correlation between increased GGT and decreased GGG RSCU in breast cancers should be investigated further.

Interest has rapidly grown in personalized mRNA vaccines for infectious diseases and cancer [53]. Codon optimization is a common technique to improve the translation efficiency of recombinant genes. Recently, it has been applied to mRNA vaccine design [53, 54]. To that end, comprehensive knowledge of codon and codon pair usage within a cancer type compared to its normal tissue would be beneficial. Based on our findings, cancers such as hepatocellular carcinoma, cholangiocarcinoma, and colorectal adenocarcinoma may be targeted by mRNA vaccines optimized to their respective codon and codon pair usage. On the other hand, tissues such as stomach, whose codon usage is not as clearly distinct from those of its respective cancers, may not benefit as greatly from this technique. However, our findings in some cancers may serve as a signature for tumor-specific codon and codon pair usage, which could be useful in downstream transcriptomic analyses and studies of translation efficiency. The degree of difference between codon usage of tumor type and its respective normal tissue remains an interesting topic that warrants further investigation. In addition, our findings of extreme variability in the level of overall codon usage change between normal and tumor tissues for 50 patients with prostate adenocarcinoma highlights the potential importance of characterizing an individual’s tissue and tumor-specific codon usage landscape when developing personalized cancer treatments, which can induce T cell responses against neoantigens unique to the patients’ mutation-derived neo-epitopes [55, 56]. If mRNA-based cancer vaccines were to be tailored for expression within the individual patient, the codon optimization schema must account for the level of codon usage difference in the individual’s normal and tumor tissue.

Finally, it is noteworthy that the degree of change in codon or codon pair usage between paired healthy and tumor tissue is associated with patient survival. One possible explanation is that aggressive tumors are likely to be more dedifferentiated or undifferentiated, leading to more divergent patterns of gene expression and consequently codon usage, a possibility that begs further investigation. These findings represent an intriguing result that implicates global codon and codon pair usage changes in the severity of disease, which should continue to be explored in future studies.

## Conclusions

In this study, we have highlighted the pertinent findings that affect our understanding of codon and codon pair usage in cancerous versus normal tissue. While some primary tumors display vastly different codon usage preferences than their tissues of origin (cholangiocarcinoma and hepatocellular carcinoma), other tissues do not appear as distinct from their respective tumors (stomach, prostate). However, a closer analysis of individual prostate cancer patients revealed that while codon usage bias in a cancer type may not appear obvious in pooled samples, there can be significant variability among patients. These findings not only add to the body of evidence for varying degrees of codon and codon pair usage bias within cancer tissues, they also have important implications for the development and optimization of personalized cancer vaccines, whose design may benefit from an understanding of the codon usage landscape of the target tumor. Furthermore, we have compiled the data that comprise the basis for these analyses into a user-friendly web interface, allowing other researchers to access pre-compiled codon and codon pair usage for 32 cancer types. Our findings provide important insights regarding the codon usage signatures of various tumors, and the associated database (https://dnahive.fda.gov/review/cancercocoputs/) [35] represents a comprehensive resource for cancer-specific codon and codon pair usage.

## Supplementary Information

**Additional file 1: Table S1.** Detailed Description of Tissue Samples Included in Each Tissue Type.

**Additional file 2: Figures.** S1-S4. All Supplementary figures.

**Additional file 3: Table S2.** MSE Values for each Cancer Type Based on Median Tissue Codon Usage.

**Additional file 4: Table S3.** Contains Table S3: Codon Usage Differences Between each Cancer and its Respective Normal Tissue.

**Additional file 5: Table S4.** Statistically Significant Codon Usage Changes in 17 Cancer Types.

**Additional file 6: Table S5.** Two-Sided Wilcoxon Signed-Rank Test P Values for All Cancers.

## Data Availability

All datasets analyzed in this study are available in our CancerCoCoPUTs database (https://dnahive.fda.gov/review/cancercocoputs/) [35]. The code used to generate tumor-specific codon and codon pair usage is available in the Cancer-CoCoPUTs GitHub Repository (https://github.com/FDA/Cancer-CoCoPUTs) [57]. TCGA transcriptomic data used to compute codon usage and associated clinical metadata were obtained from GDC [36].

## Abbreviations

TCGA: The Cancer Genome Atlas
IDC: Invasive ductal carcinoma, breast
ILC: Invasive lobular carcinoma, breast
IDLC: Invasive mixed ductal and lobular carcinoma, breast
mRNA: Messenger ribonucleic acid
RSCU: Relative synonymous codon usage
BRCA1: Breast cancer type 1 susceptibility protein
BRCA2: Breast cancer type 2 susceptibility protein
APC: Adenomatous polyposis coli
EGFR: Epidermal growth factor receptor
SNP: Single nucleotide polymorphism
KRAS: KRAS proto-oncogene
HER2: Human epidermal growth factor receptor 2
tRNA: Transfer ribonucleic acid
Pro: Proline
Gly: Glycine
GDC: Genome Data Commons
TCC: Transitional cell carcinoma
SCC: Squamous cell carcinoma
CDS: Coding sequence
TPM: Transcripts per million
MSE: Mean squared error
PCA: Principal component analysis
LUAD: Lung adenocarcinoma
LUSC: Lung squamous cell carcinoma
Arg: Arginine
Cys: Cysteine
Ile: Isoleucine
GTEx: Genotype-Tissue Expression Project
PC1: Principal component 1
PC2: Principal component 2
