# Supplementary material for: Distinct signatures of codon and codon pair usage in 32 primary tumor types in the novel database CancerCoCoPUTs for cancer-specific codon usage
Source: Genome Med. 2021 Jul 28;13:122. doi: 10.1186/s13073-021-00935-6 (PMC8317675; doi:10.1186/s13073-021-00935-6)
Supplement: Supplementary file 2 — Additional file 2: Figures. S1-S4. All Supplementary figures. [file 13073_2021_935_MOESM2_ESM.pdf]

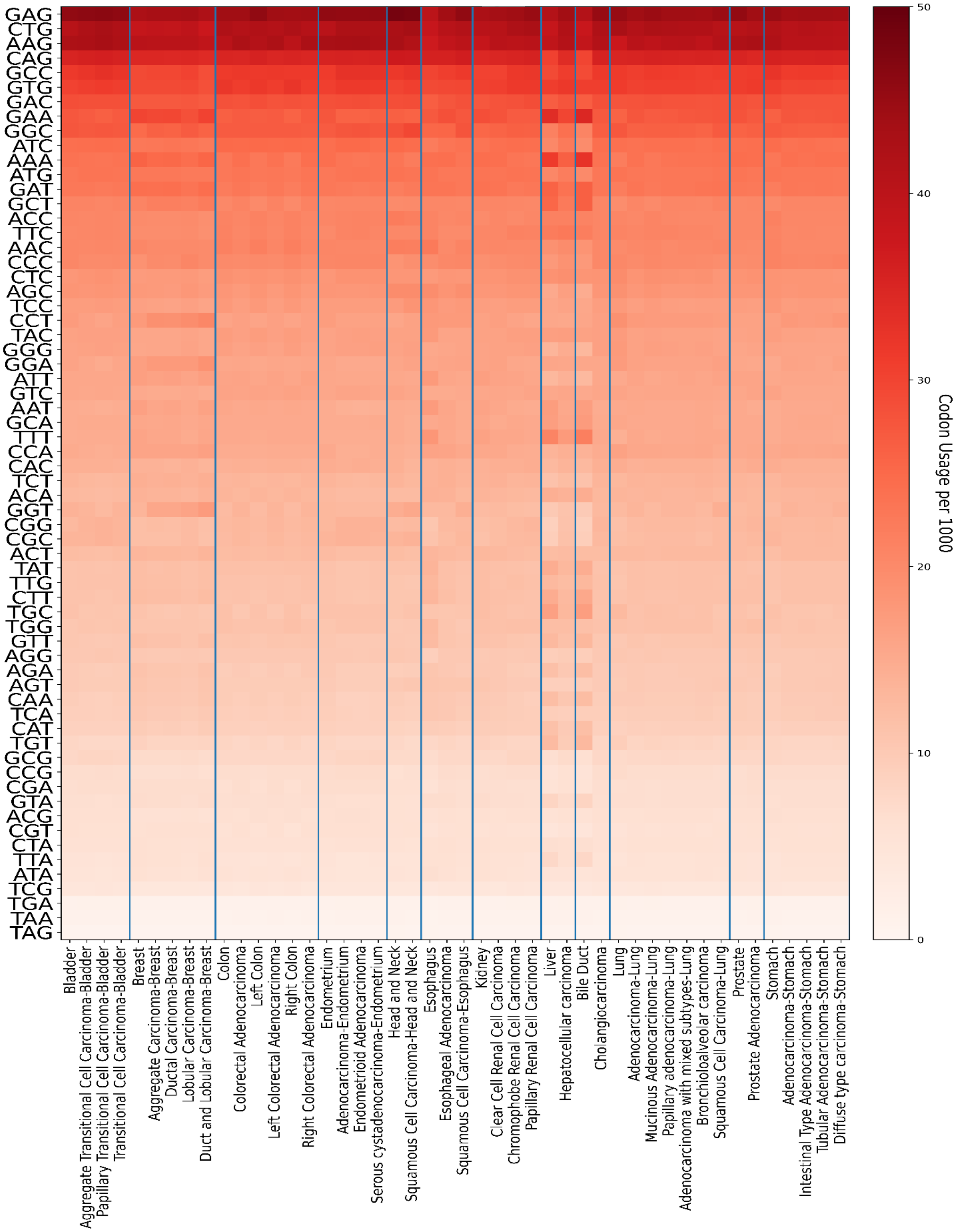

Figure S1: Codon Usage per One Thousand for All Tissue Types

Heatmap showing codon usage for all primary tumor and normal tissue types evaluated in this study. Light values represent low usage while darker red values indicate higher usage. Normal tissue types are grouped with all associated primary tumor types. Codons along y axis are ordered based on median usage across all normal tissue types with highly used codons appearing at the top. This image is an enlarged version of the heatmap shown in figure 1C.

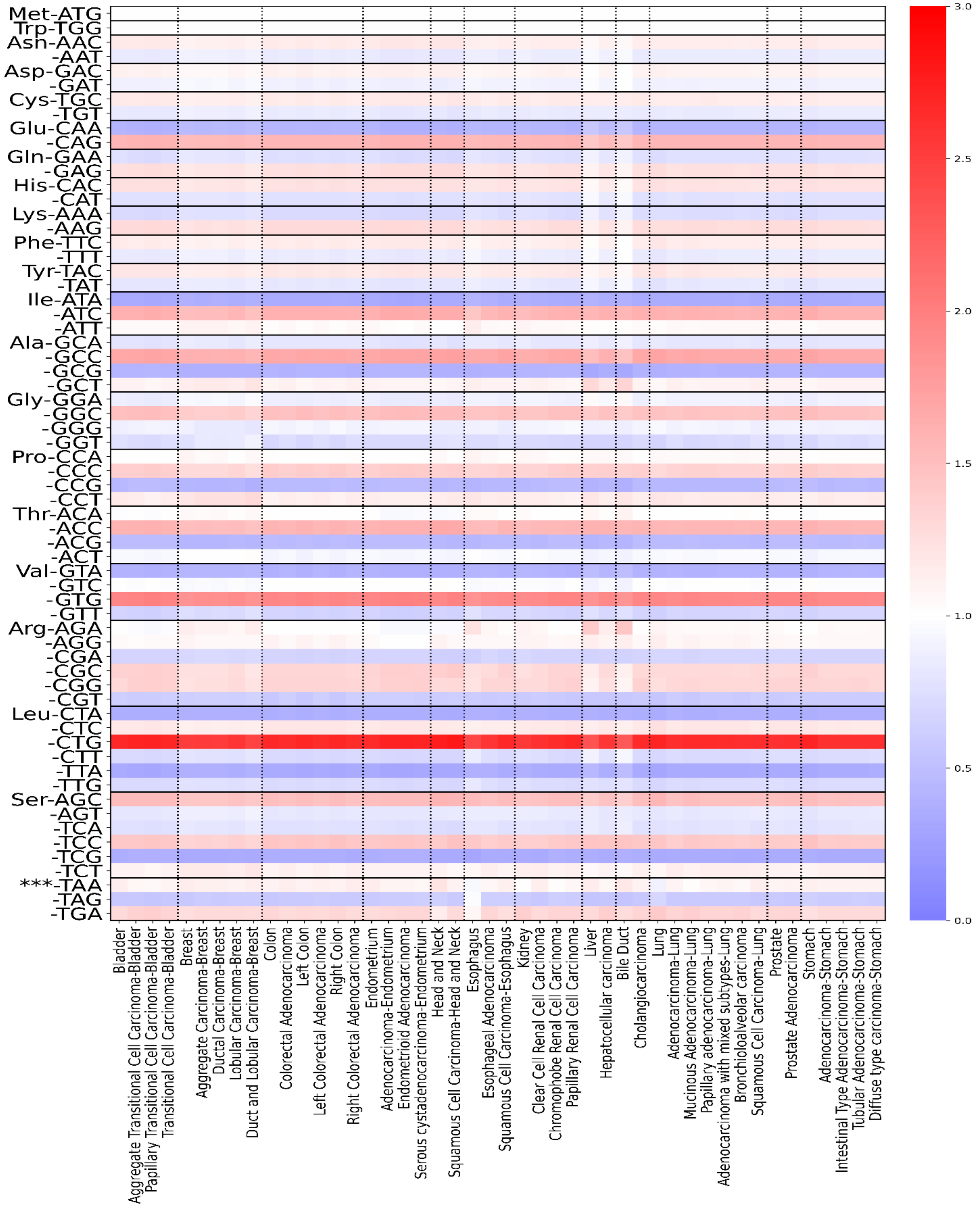

Figure S2: Relative Synonymous Codon Usage for All Tissue Types

Heatmap showing relative synonymous codon usage (RSCU) for all primary tumor and normal tissue types evaluated in this study. Red represents RSCU > 1 while blue represents RSCU < 1 and white represents RSCU = 1. Synonymous codons with red values are preferred over synonymous codons with blue values. Horizontal black lines separate synonymous codon groups. Normal tissue types are grouped with all associated primary tumor types and vertical dashed lines separate these tissue groups.

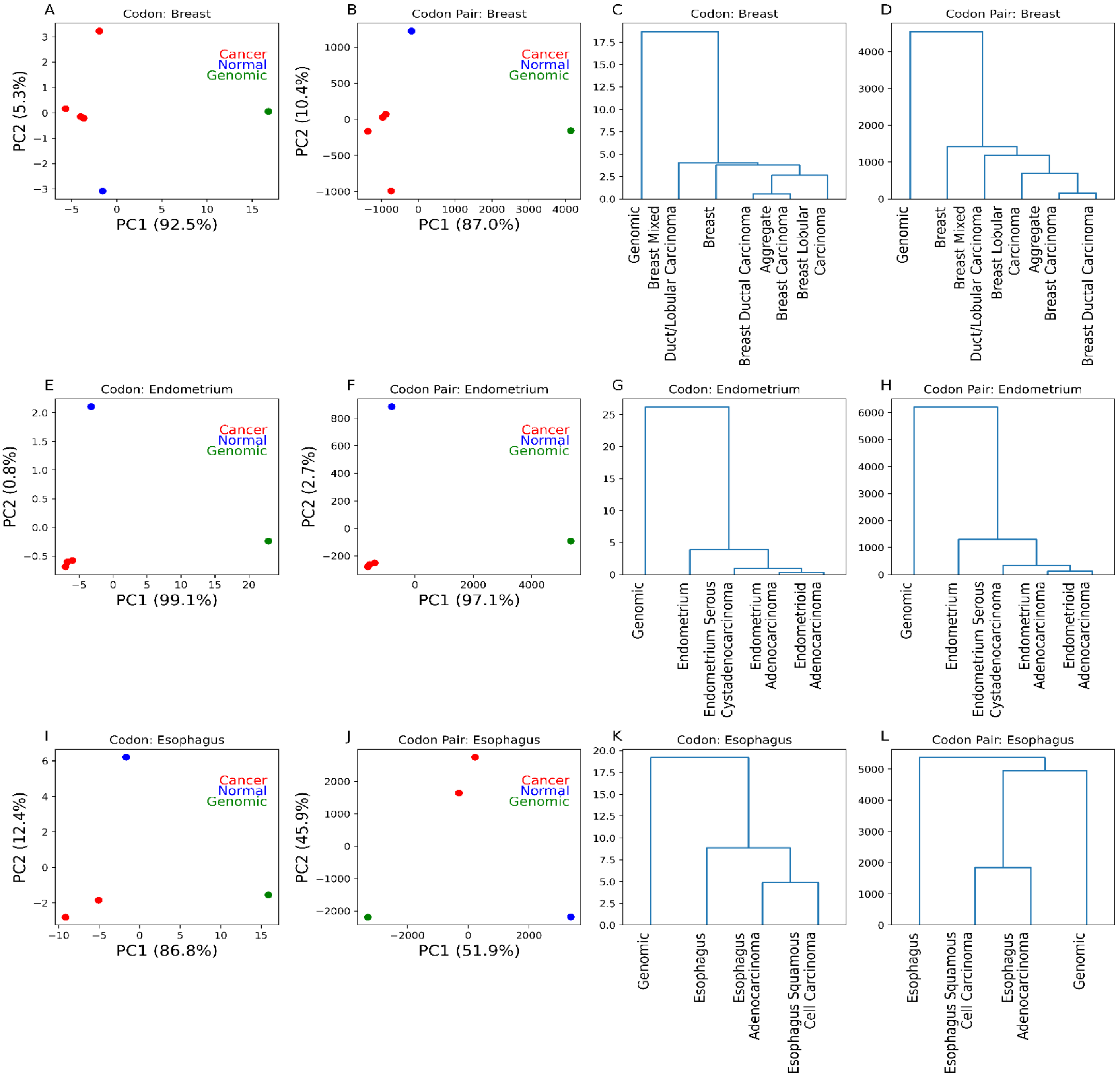

Figure S3: Aggregate Analysis of Different Cancers from Identical Tissues of Origin

S3A, B: Principal component analysis for codon (A) and codon pair (B) usage in normal breast tissue, invasive ductal carcinoma (IDC), invasive lobular carcinoma (ILC) and invasive mixed ductal and lobular carcinoma (IDLC). Genomic codon and codon pair usage values are not transcriptome weighted.
S3C, D: Euclidean distance dendrograms based on tissue-specific codon usage (C) or codon pair usage (D)
S3E, F: Principal component analysis for codon (E) and codon pair (F) usage in normal endometrium tissue, endometroid adenocarcinoma, serous cystadenocarcinoma and adenocarcinoma of the endometrium. Genomic codon and codon pair usage values are not transcriptome weighted.
S3G, H: Euclidean distance dendrograms based on tissue-specific codon (G) or codon pair (H) usage
S3I, J: Principal component analysis for codon (I) and codon pair (J) usage in normal esophageal tissue, esophageal adenocarcinoma and esophageal SCC. Genomic codon and codon pair usage values are not transcriptome weighted.
S3K, L: Euclidean distance dendrograms based on tissue-specific codon usage (K) or codon pair usage (L)

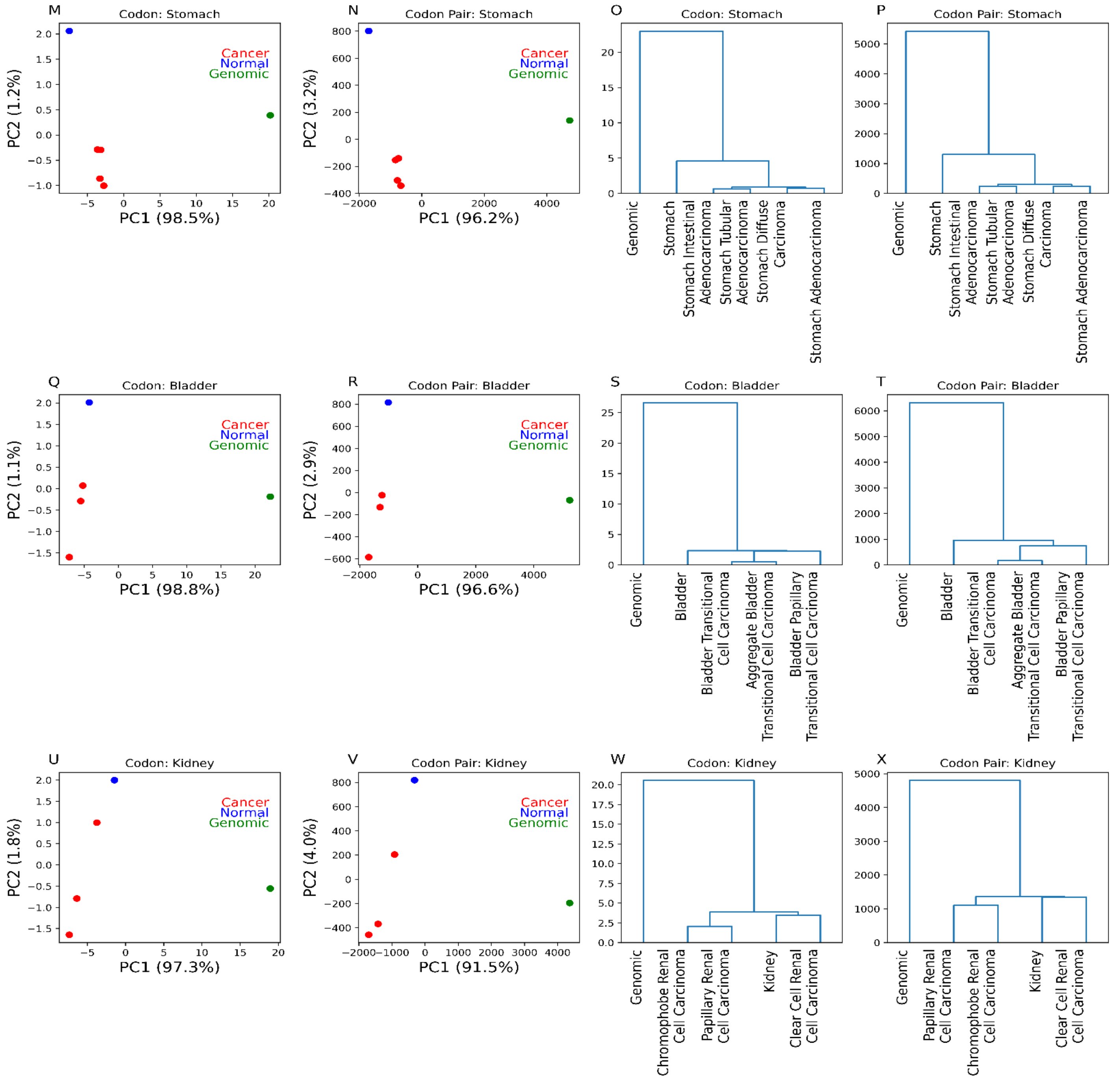

Figure S3 (continued): Aggregate Analysis of Different Cancers from Identical Tissues of Origin

S3M, N: Principal component analysis for codon (M) and codon pair (N) usage in normal gastric tissue, gastric adenocarcinoma, gastric intestinal adenocarcinoma, gastric tubular adenocarcinoma and gastric diffuse carcinoma. Genomic codon and codon pair usage values are not transcriptome weighted.

S3O, P: Euclidean distance dendrograms based on tissue-specific codon usage (O) or codon pair usage (P)

S3Q, R: Principal component analysis for codon (Q) and codon pair (R) usage in normal bladder tissue, aggregate transitional cell carcinoma, papillary transitional cell carcinoma and transitional cell carcinoma of the bladder. Genomic codon and codon pair usage values are not transcriptome weighted.

S3S, T: Euclidean distance dendrograms based on tissue-specific codon usage (S) or codon pair usage (T)

S3U, V: Principal component analysis for codon (U) and codon pair (V) usage in normal kidney tissue, chromophobe, papillary and clear cell renal cell carcinomas. Genomic codon and codon pair usage values are not transcriptome weighted.

S3W, X: Euclidean distance dendrograms based on tissue-specific codon usage (W) or codon pair usage (X)

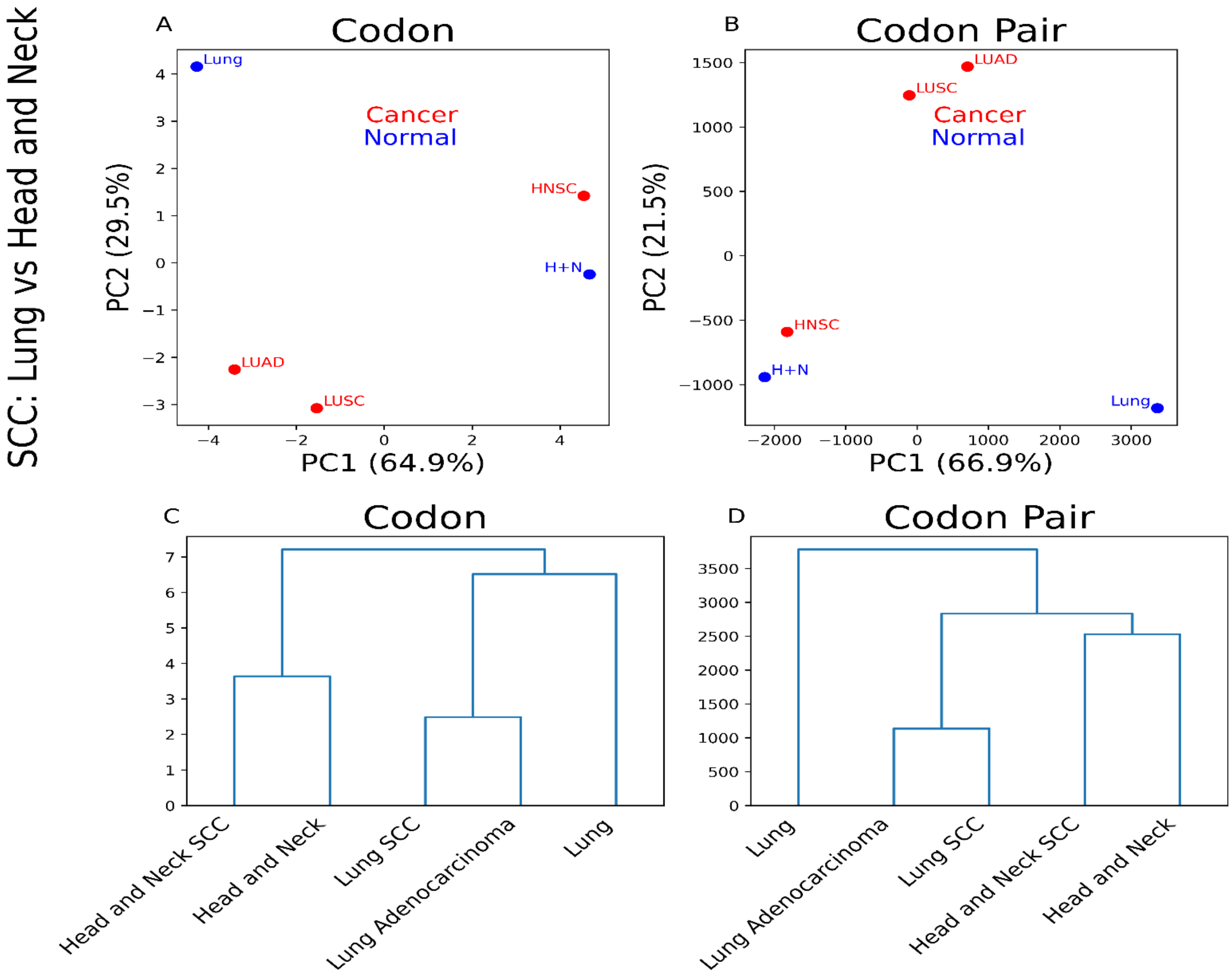

Figure S4: Comparing Squamous Cell Carcinoma from Different Origins

S4A, B: Principal component analysis for normal lung tissue (Lung), lung squamous cell carcinoma (LUSC), lung adenocarcinoma (LUAD), normal head and neck (H+N) and squamous cell carcinoma of the head and neck (HNSC) based on codon usage (A) and codon pair usage (B).

S4C, D: Euclidean distance dendrogram for lung and head and neck tissues based on codon usage (C) and codon pair usage (D).
